# Moderate Calorie Restriction Enhances Hepatic Glucagon Sensitivity in Aged Mice

**DOI:** 10.1093/geroni/igab046.2556

**Published:** 2021-12-17

**Authors:** Stephen Hutchison, Anastasiia Vasileva, Tyler Marx, Samantha Slavin, Jennifer Stern

**Affiliations:** University of Arizona, Tucson, Arizona, United States

## Abstract

Chronic calorie restriction (CR) without malnutrition delays the onset of aging, extends lifespan, and improves metabolic function in many species. These CR-induced benefits have largely concentrated on the role of insulin signaling, while ignoring its counter-regulatory hormone, glucagon. Like insulin, hyperglucagonemia and decreased glucagon sensitivity are associated with impaired glucose homeostasis and decreased longevity. Conversely, activation of target molecules downstream of glucagon signaling such as AMPK and FGF21 are known to ameliorate these age-related impairments in metabolic function. To investigate the potential role of glucagon receptor signaling in CR-induced improvements in aging, we have implemented a moderate 15% CR in the mouse. Our studies show that a 15% calorie restriction initiated at 4 months of age enhances hypoglycemia-stimulated glucagon secretion (P<.01) and decreases basal serum glucagon (P<.01), while having no effect on glucagon receptor expression at the liver in 26-month-old mice. Consistent with enhanced hepatic glucagon sensitivity, CR increases glucagon-stimulated hepatic cyclic AMP production (P<.05). Glucagon is a primary regulator of AMPK activation and FGF21 release, both of which have been proposed as key molecules to account for CR-induced benefits to aging. CR increases both hepatic AMPK activation (P<.05) and FGF21 mRNA expression (P<.05). Additionally, CR reduces hepatic lipid accumulation (P<.05), and decreases fasting respiratory quotient (P<.001), indicating an increase in lipid oxidation. Our studies demonstrate that a moderate (15%) CR regimen enhances glucagon sensitivity and decreases hepatic lipid accumulation in aged mice. Thus, we propose glucagon signaling as a mediator of CR-induced improvements in aging.

